# Fission yeast spindle dynamics and chromosome segregation fidelity show distinct thermosensitivity.

**DOI:** 10.17912/micropub.biology.001048

**Published:** 2024-01-09

**Authors:** Zachary Chaba, Ishutesh Jain, Phong T. Tran

**Affiliations:** 1 Cupertino High School, Cupertino, CA 95014, USA; 2 Simons Centre for the Study of Living Machines, National Centre for Biological Sciences - TIFR, Bangalore 560065, India; 3 Institut Curie, PSL Université, Sorbonne Université, CNRS UMR 144, Paris 75005, France; 4 University of Pennsylvania, Department of Cell and Developmental Biology, Philadelphia, PA 19104, USA

## Abstract

Cellular processes rely on proteins with temperature-dependent stability and activity. While thermosensitivity in biological networks is well-explored, the effect of temperature on complex mechanochemical assemblies, like the spindle, is rarely studied. We examined fission yeast spindle dynamics and chromosome segregation from 15⁰C to 40⁰C. Our findings reveal that these parameters follow U-shaped temperature-dependent curves but reach their minima at different temperatures. Specifically, spindle dynamics peak around 35⁰C, whereas chromosome segregation defects are minimized at 25⁰C. This suggests a scenario in which mitotic errors are tolerated to expedite rapid cell cycle progression.

**Figure 1. Fission yeast spindle dynamics and chromosome segregation fidelity follow distinct temperature-dependent U-shaped curves:**
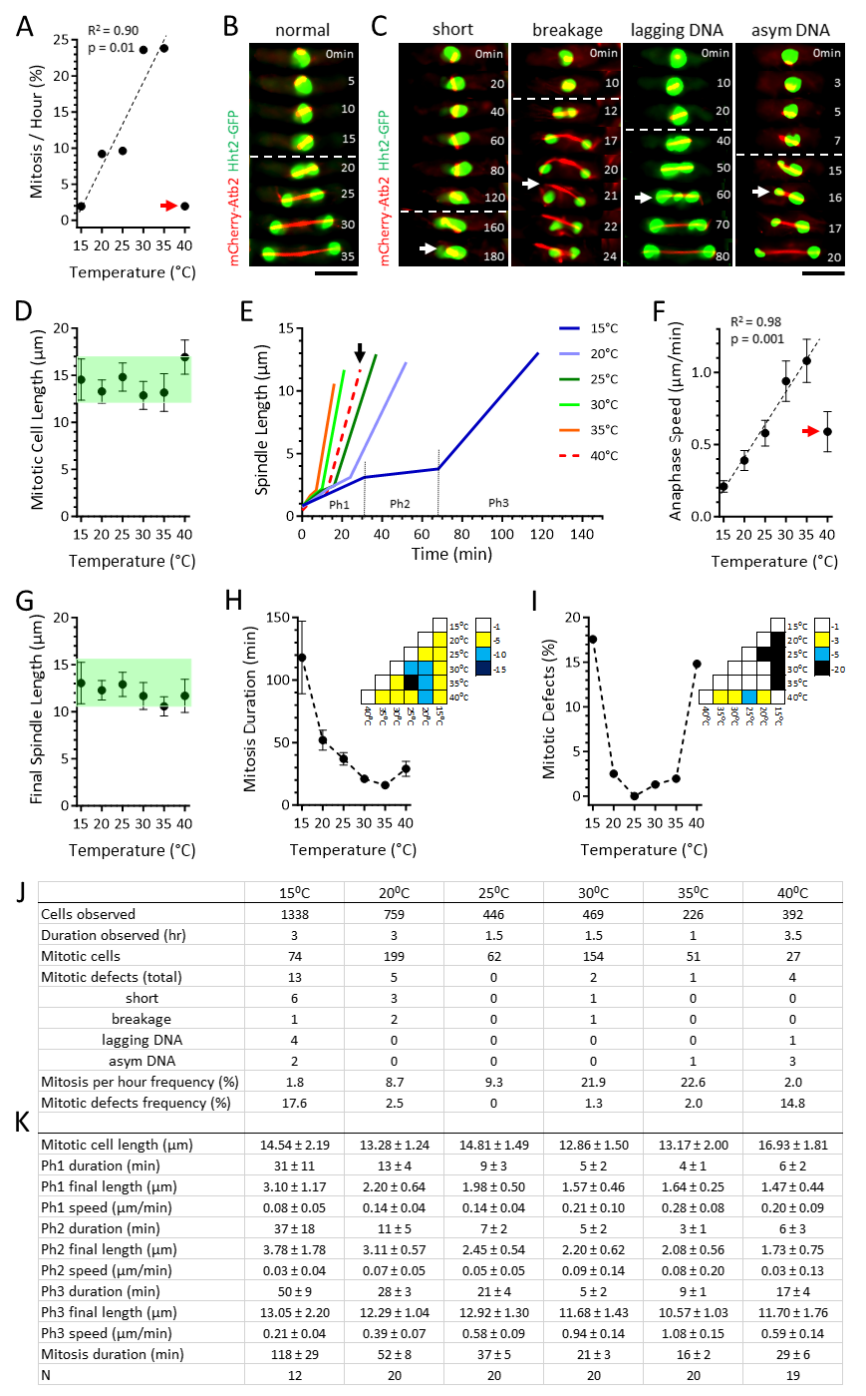
**A.** Plot of the observed frequency of mitotic cells per hour versus temperature. From 15⁰C to 35⁰C the mitotic frequency positively correlates with increasing temperature. However, at 40⁰C the mitotic frequency decreases dramatically (red arrow). Regression analysis coefficient of determination R ^2^ and p-value are shown. **B.** A ‘normal’ mitotic cell. Time-lapsed images of a wild-type cell expressing mCherry- Atb2 (tubulin) and Hht2 -GFP (histone) undergoing mitosis at 25⁰C. White dashed line delineates the start of anaphase B, where sister chromosomes are separated to the pole as the spindle dramatically elongates. Spindle assembly and elongation correspond to the typical error-free symmetrical chromosome segregation, where equal chromosome masses are present in daughter cells. Scale bar, 5 μm. **C.** Representative defective mitotic cells: ‘short’ highlights a 15⁰C spindle that failed to elongate over an abnormally long period of time (white arrow, 180 min time point); ‘breakage’ highlights a 30⁰C spindle that buckled and broke (white arrow, 21 min time point), resulting in defective chromosome positioning; ‘lagging DNA’ highlights a 15⁰C spindle exhibiting chromosome lagging during anaphase, resulting in defective chromosome segregation (white arrow, 60 min time point); ‘asym DNA’ highlights a 35⁰C spindle exhibiting asymmetric or unequal chromosome mass separation at anaphase, resulting in daughter cells with abnormal DNA content. White dashed line delineates the start of anaphase B. Scale bar, 5 μm. **D.** Plot of mitotic cell length versus temperature. Shown are mean ± standard deviation. Green shade highlights similarity among the values, except at 40⁰C. **E.** Plot of spindle length versus time. Shown are mean spindle length at respective temperature. Spindle dynamics follow the typical 3-phase kinetics. Representative phases are shown for 15⁰C: Ph1, prophase; Ph2, metaphase-anaphase A; and Ph3, anaphase B. Note that spindle dynamics trend progressively faster with increasing temperature, except at 40⁰C (black arrow). **F.** Plot of anaphase elongation speed versus temperature. Shown are mean ± standard deviation. From 15⁰C to 35⁰C the speed positively correlates with increasing temperature. However, at 40⁰C the speed decreases dramatically (red arrow). Regression analysis coefficient of determination R ^2^ and p-value are shown. **G.** Plot of final spindle length versus temperature. Shown are mean ± standard deviation. Green shade highlights similarity among the values. **H.** Plot of mitotic duration versus temperature. Shown are mean ± standard deviation. Dashed line highlights trend of the values. (Inset) p-values obtained using the t-test across temperatures. The color bar indicates the scale of p-values, represented as powers of 10. **I.** Plot of frequency of spindle defects (cumulative sum of different defects shown in Fig. 1B) versus temperature. Dashed line highlights trend of the values. (Inset) p-values obtained using the ꭓ ^2^ -test across temperatures. The color bar indicates the scale of p-values, represented as powers of 10. **J.** Table of cumulative observed cells, mitotic cells, and mitotic defects at different temperatures. Mitosis per hour frequency is calculated as % = Mitotic cells/Cells observed/Duration observed. Mitotic defects frequency was calculated as % = Mitotic defects/Mitotic cells. **K.** Table of the 3-phase spindle dynamic parameters at different temperatures. Ph1, prophase; Ph2, metaphase-anaphase A; Ph3, anaphase B. Shown are mean ± standard deviation.

## Description

Temperature can affect cellular functions in a multitude of ways. First, the reaction rate of enzymes is temperature-dependent, with an increase in temperature enhancing the reaction rate. Second, temperature affects the viscosity of cytoplasm; lower temperatures reduce the fluidity of cytoplasm and alter its transport properties. Third, extreme temperature changes can lead to significant rearrangements of protein conformations, such as unfolding or mixing, resulting in substantial behavioural changes. Due to these complex effects, understanding how temperature impacts physiology has remained a topic of interest. Extensive research has been conducted on the biological adaptation of protein function to temperature changes (Knapp and Huang, 2022) . These studies suggest that biological systems operate within a narrow ‘optimal’ temperature range, where reaction rates follow Arrhenius law. Beyond this range, protein destabilization necessitates an adaptive response to sustain function. However, it remains unclear whether these principles can be extended to large supramolecular assemblies, such as spindle apparatuses formed during mitosis. In this study, we aim to investigate fission yeast spindle assembly dynamics and chromosome segregation in response to temperatures ranging from 15⁰C to 40⁰C.

A wild-type fission yeast strain expressing endogenous levels of mCherry- Atb2 (tubulin, microtubule marker) and Hht2 -GFP (histone, DNA marker) were first grown overnight at 25⁰C. Cells were then transferred onto an agarose slide for live-cell imaging using a spinning disk confocal microscope, which was preset to different temperatures: from 15⁰C to 40⁰C, with 5⁰C increments (see Methods). We restricted our study to this temperature range due to two key factors: 1) microtubules are known to depolymerize below 15⁰C in fission yeast (Velve-Casquillas et al., 2011), preventing spindle formation; and 2) cell viability is compromised above 40⁰C (Forsburg and Rhind, 2006) . We allowed approximately 30 minutes for the cells to acclimate to each specific temperature before acquiring images. We observed a linear increase in the frequency of mitosis between 15⁰C and 35⁰C, with the percentage of cells undergoing mitosis per hour rising from approximately 2% to 24% ( Fig. 1A, 1J). However, at 40⁰C, only about 2% of cells underwent mitosis per hour. The linear increase in mitotic frequency can be explained by the temperature-dependent changes in reaction rate kinetics, following Arrhenius law. The low mitotic frequency at 40⁰C is likely due to a heat-shock response, as we did not observe any mitotic cells during the first two hours of observation at this high temperature. Instead, heat-shocked cells continued to grow, resulting in slightly longer cell lengths prior to mitosis ( Fig. 1D ).

Fission yeast spindle dynamics and chromosome segregation progress through three phases: Phase 1 (prophase), Phase 2 (metaphase/anaphase A), and Phase 3 (anaphase B), each characterized by distinct spindle elongation kinetics and chromosome positions (Nabeshima et al., 1998) . At all temperatures, we observed that the majority of cells displayed these three phases in a typical manner ( Fig. 1E, 1K), with the chromosome mass equally separated during anaphase ( Fig. 1B ). We noted that, except at 40⁰C, the rates of spindle elongation for the three phases, especially Phase 3 (anaphase B), exhibited a linear correlation with increasing temperature ( Fig. 1E, 1F, 1K). However, parameters such as cell length at mitosis or final spindle length appeared similar at all temperatures ( Fig. 1D, 1E, 1G, 1K). Thus, while spindle elongation velocity depends on temperature, following Arrhenius law, certain outcomes, such as final spindle length and cell length, are independent of temperature, suggesting that these functional parameters robustly respond to temperature changes to maintain their required functions.

While the majority of cells behaved as expected of wild-type at different temperatures, we did observe instances of spindle assembly and chromosome segregation defects, particularly at the extremes of temperatures ( Fig. 1C, 1J). We categorized four types of defects: sustained ‘short’ spindle resulting in failed mitosis; spindle ‘breakage’ or collapse resulting in chromosome malposition; ‘lagging DNA’ during anaphase resulting in defective chromosome segregation, and ‘asymmetrical DNA’ at anaphase resulting in unequal chromosome distribution in daughter cells ( Fig. 1C, 1J). We observed that at the low range of temperatures, the short spindle phenotype dominates ( Fig. 1J, 15⁰C); and at the high range of temperatures, the asymmetric DNA phenotype dominates ( Fig. 1J, 40⁰C). This indicates that low and high temperatures have distinct effects on spindle assembly and chromosome segregation: at low temperature, microtubule dynamics and thus spindle assembly may be affected; and at high temperature, kinetochore-microtubule attachment and thus chromosome segregation may be affected. As spindle assembly precedes chromosome segregation, defects occurring during spindle assembly may consequently and additionally affect chromosome segregation ( Fig. 1J, 15⁰C).

To better visualize the effect of temperature on spindle assembly dynamics and chromosome segregation, we plotted mitosis duration versus temperature, and mitotic defects (i.e., the combined four types of defects measured) versus temperature ( Fig. 1J ). Both mitosis duration and mitotic defects showed unique U-shaped dependency on temperature ( Fig. 1H, 1I). The U-shaped mitosis duration curve reached its minimum at 35⁰C ( Fig. 1H ). Surprisingly, the U-shaped mitotic error curve showed a minimum at 25⁰C ( Fig. 1I ). These results indicate that the optimal temperature for spindle assembly dynamics is not the same as the optimal temperature for minimizing chromosome segregation defects in fission yeast. We suggest that this reflects a situation where mitotic defects are tolerated to allow for fast cell cycle progression.

Mitosis has long been known to exhibit extreme sensitivity to temperature (Rao and Johnson, 1970) . Building upon this established observation, our study delves into the intricate relationship between temperature and spindle assembly dynamics and chromosome segregation. Consistent with prior findings (Wu et al., 2003) , we also noted temperature-dependent changes in spindle elongation kinetics and mitotic frequency. These discoveries align with the principles of Arrhenius law, which governs reaction rates in biological systems. Notably, our results unveil a delicate equilibrium where specific functional parameters, such as final spindle and cell length, remain remarkably impervious to temperature variations, thus ensuring the robustness of cellular functions. Moreover, our exploration of mitotic defects underscores the complexity of biological responses to temperature extremes, revealing distinct optimal temperature ranges for spindle dynamics and chromosome segregation. This divergence may reflect a tolerance for mitotic defects, even at the risk of cell death, to attain a higher growth rate. Future research in this direction holds the potential to illuminate the broader implications of temperature on cell biology and physiology , particularly for poikilotherm organisms, such as the fission yeast, which cannot regulate their internal temperature, versus homeotherms organisms, which do regulate their body temperature.

## Methods

Yeast strains and media:

We employed standard yeast genetics techniques to create and maintain the strain utilized in this study (Moreno et al., 1991). Typically, cells were cultured on agar plates containing YE5S media at a temperature of 25⁰C.

For microscopy experiments, a small number of cells were inoculated into liquid YE5S medium the night before the experiment. The culture was then incubated at 25⁰C with continuous shaking overnight until it reached an optical density OD _600nm_ ≈ 0.5 absorbance units. At this point, the cells were harvested for imaging. The cells were mounted in YE5S media within 2% agar using custom-made PDMS chambers, as previously described (Costa et al., 2013) .

Microscopy:

Imaging was conducted using a spinning disk confocal microscope setup (Tran et al., 2004) . Specifically, we utilized a Nikon Eclipse Ti2 inverted microscope equipped with a Nikon CFI Plan Fluor 100x/1.4 NA objective lens, a Nikon Perfect Focus System (PFS), a Mad City Labs integrated Nano-View XYZ micro- and nano-positioner, a Yokogawa Spinning Disk CSU-X1 unit, a Photometrics Evolve EMCCD camera, and a Gataca Systems solid-state laser unit with 488 nm (100 mW) and 561 nm (100 mW) lines, controlled by Molecular Devices MetaMorph 8.0 software. The microscope was enclosed inside a Life Imaging Services thermal box, with temperature controlled via forced air from the thermal Cube. The temperature can be maintained stably at the set point ± 1⁰C.

To capture the images, we acquired z-stacks consisting of 7 focal planes spaced 1 µm apart in the GFP (Exposure time: 100 ms) and mCherry (Exposure time: 100 ms) channels. A corresponding brightfield image (Exposure: 50 ms) was acquired to visualize cells. The stacks were acquired through time-lapse, depending on the specific temperature, between 1 to 3.5 hr, at 0.5 min to 2 min intervals.

Analysis:

Maximun-projection time-lapsed movies were visualized and analysed by ImageJ/FIJI (Schindelin et al., 2012) . Statistical analysis was performed using Google Sheets. Plots were generated using GraphPad Prism.

## Reagents

Strain used: TP. 3640 h+ mCherry- Atb2 :HygR Hht2 -GFP: Ura4
